# Pharmacokinetic and Pharmacodynamic Modeling of Clonidine and Midazolam for Sedation in Pediatric Intensive Care

**DOI:** 10.1111/pan.70050

**Published:** 2025-10-04

**Authors:** Maddlie Bardol, Yucheng Sheng, Manuel Baarslag, Adriana Ceci, Frank Dörje, Mari‐Liis Ilmoja, Peter Larsson, Per‐Arne Lönnqvist, Tuuli Methsvat, Pavla Pokorna, Wolfgang Rascher, Joost van Rosmalen, Michael Schroth, Alessandra Simonetti, Dick Tibboel, Irmgard Toni, Catherine Tuleu, Thomas M. K. Völkl, Brian J. Anderson, Stefan Wimmer, Joseph F. Standing, Antje Neubert, CloSed Consortium

**Affiliations:** ^1^ Institute of Child Health University College London London UK; ^2^ Pharmetheus AB Uppsala Sweden; ^3^ Department of Pharmaceutics, School of Pharmacy University College London London UK; ^4^ Erlangen/Nürnberg Erlangen, Germany, Intensive Care and Department of Pediatric Surgery Erasmus MC Rotterdam the Netherlands; ^5^ Department of Research Gianni Benzi Pharmacological Research Foundation Bari Italy; ^6^ Pharmacy Department Universitätsklinikum Erlangen Erlangen Germany; ^7^ Department of Anaesthesiology and Intensive Care Tallinn Children's Hospital Tallinn Estonia; ^8^ Department of Pediatric Perioperative Medicine and Intensive Care Karolinska University Hospital Stockholm Sweden; ^9^ Department of Microbiology University of Tartu Tartu Estonia; ^10^ Department of Pediatrics General Faculty Hospital Prague, First Faculty of Medicine Charles University Prague Czech Republic; ^11^ Department of Physiology and Pharmacology Karolinska University Hospital Stockholm Sweden; ^12^ Department of Paediatrics and Adolescent Medicine Friedrich‐Alexander‐Universität Erlangen‐Nürnberg (FAU), Faculty of Medicine, Universitätsklinikum Erlangen Erlangen Germany; ^13^ Department of Biostatistics Erasmus MC Rotterdam the Netherlands; ^14^ Department of Epidemiology Erasmus MC Rotterdam the Netherlands; ^15^ Cnopf Children's Hospital Nuremberg Nuremberg Germany; ^16^ Bambino Gesù Children's Hospital Clinical Trial Center Rome Italy; ^17^ School of Pediatrics University of Rome “Tor Vergata” Rome Italy; ^18^ Department of Paediatrics and Adolescent Medicine KJF Klinikum Josefinum Augsburg Germany; ^19^ Department of Anaesthesiology University of Auckland Auckland New Zealand

**Keywords:** children, pediatrics, pharmacodynamics, pharmacokinetics, pharmacology, sedation

## Abstract

**Background:**

Clonidine and midazolam are routinely used in the pediatric intensive care unit for pain and sedation management, but target concentration and optimal dose are poorly defined for both drugs. The CloSed study is a multicenter, double‐blind, randomized, active‐controlled noninferiority trial with a 1:1 randomization between clonidine and midazolam.

**Aims:**

Data from the prematurely terminated CloSed trial were used to study the population pharmacokinetic–pharmacodynamic relationships for clonidine and midazolam to inform the optimal use of both drugs in mechanically ventilated children.

**Methods:**

Twenty‐eight patients (0–6 years) were included; 13 received midazolam, and 15 received clonidine. Morphine was administered to all patients as background analgesia. A total of 317 and 306 observed COMFORT‐B scores for midazolam and clonidine, respectively, were available to build the pharmacokinetic–pharmacodynamic model. Pharmacokinetic models were developed using findings from previously published pharmacokinetic studies to augment the trial data. A one‐compartment model described clonidine pharmacokinetics, while a single compartment for midazolam and its metabolite described its pharmacokinetics. A joint inhibitory sigmoid model that included a postanesthesia effect was used to describe the concentration–effect relationship, using the COMFORT‐B score as the pharmacodynamic endpoint.

**Results:**

The final models adequately described the observed data. Simulations based on the final models showed that a clonidine dose of 4 μg/kg loading dose followed by a 3 μg/kg/h infusion, and a midazolam dose of 200 μg/kg loading dose followed by a 200 μg/kg/h infusion would be required to achieve adequate sedation.

**Conclusion:**

The CloSed data suggest that higher doses of clonidine and midazolam than are commonly used in clinical practice should be considered to provide adequate sedation in critically ill children.

## 1 Introduction

There are relatively few pharmacokinetic–pharmacodynamic studies involving sedatives conducted in children, even though these drugs are routinely used in the *pediatric intensive care unit* (PICU) for pain and sedation management [1]. The CloSed study (CLonidine compared with midazolam for Sedation of children in the intensive care unit) was a trial that aimed to compare clonidine with midazolam for sedation in the PICU [2].

Midazolam is a short‐acting benzodiazepine that provides anxiolysis, sedation, amnesia, and muscle relaxation. It is mainly used in combination with a strong analgesic drug, such as an opioid [3]. Midazolam is the sedative most commonly prescribed in the intensive care unit because it presents additional advantages such as seizure control and anterograde amnesia [4]. The common adverse effects of midazolam include respiratory depression, tolerance, dependence, and withdrawal syndrome. Midazolam undergoes hydroxylation by CYP3A4 and CYP3A5 to form two metabolites, 1‐hydroxymidazolam and 4‐hydroxymidazolam, that contribute to 10% of the sedative effect of the drug [4].

Clonidine is an alpha‐2‐adrenergic receptor agonist that has analgesic effects and reduces discomfort and agitation in children [5]. Clonidine also limits the stress response by suppressing the increase of both sympathetic outflow and vasoconstrictors, preventing organ failure after surgery [6]. The drug is also associated with neuroprotective effects [7]. Adverse effects include bradycardia and hypotension; however, the incidence of these adverse effects is low. Clonidine has a long elimination half‐life (17 h in neonates) [8]. A loading dose is required to reach the therapeutic steady state concentrations in less than 24 h. Clonidine dose is commonly reduced when administered with other sedatives (e.g., midazolam) or analgesics (e.g., morphine), although the nature of these pharmacokinetic or pharmacodynamic interactions remains poorly defined [9].

Running comparative clinical trials for sedation in children is challenging because patient recruitment can be difficult within the allocated time and funding envelope, even with multinational recruitment. In addition, the frequency and severity of adverse events, as well as rescue medications needed, are often different in both arms, making it challenging to compare the efficacy between the drugs [10]. The double‐blind CloSed trial [2] was originally designed to generate data necessary to obtain a Paediatric Use Marketing Authorisation (PUMA) for clonidine use in PICU. However, the trial was prematurely terminated on the advice of the Data Safety Monitoring Committee and funder due to slow recruitment leading to poor prospects for meeting the primary endpoint, which was to assess the noninferiority of continuous intravenous clonidine compared to midazolam [S1].

A secondary objective of the CloSed trial was to develop pharmacokinetic–pharmacodynamic (PKPD) models using pain and sedation scores as efficacy endpoints for clonidine and midazolam. This paper presents the modeling work done using the data collected to inform the optimal use of clonidine and midazolam in mechanically ventilated children.

## 2 Methods

### 2.1 Patient Recruitment

The CloSed trial (EudraCT: 2014–003582‐24, Clinicaltrials.gov: NCT02509273) was a double‐blind, multicenter, phase III randomized controlled trial with two parallel groups (clonidine vs. midazolam) [2]. The trial ran from May 2016 to October 2018. Ethical approval was obtained for each of the five participating European centers.

A sample size of 300 patients was originally planned. Children were included if the following criteria were met: Age younger than 18 years, expected admission to the PICU, expected indication for mechanical ventilation, and need for continuous sedation for at least 24 h. Informed consent from the parent and, if feasible, patient assent were required. The exclusion criteria included a gestational age younger than 34 weeks, severe organ insufficiencies, brain injuries, acute asthma, severe bradycardia, and arterial hypertension. Patients given sedation for more than 72 h prior to the screening were also excluded.

### 2.2 Drug Administration, Sampling, and Assay

The investigational medical product (IMP) (clonidine or midazolam) was administered for a maximum of 7 days. The starting dose for clonidine was 2 μg/kg loading dose (15 min) followed by 1 μg/kg/h continuous infusion. The midazolam starting dose was 200 μg/kg, followed by 100 μg/kg/h. The doses were halved in neonates with postnatal age (PNA) younger than 28 days. Trial IMP vials were prepared in three strengths (low, medium, and high) such that the volume to be administered was identical for each drug to maintain blinding. IMP was manufactured by the Hospital Pharmacy of the Universitätsklinikum Erlangen, Germany [11].

The maintenance infusion rates for midazolam and clonidine were adjusted using a dosing algorithm [S2] based on the sedation levels assessed using both the COMFORT‐B score and the Nurses’ Interpretation of Sedation Score (NISS).

In addition to the IMP, all patients received a continuous infusion of morphine for analgesia. Patients who had not been given morphine at trial enrollment received a bolus of 100 μg/kg. Infusion was started for all patients at 5 μg/kg/h. If the numeric rating scale (NRS) was ≥ 4, another 10 μg/kg bolus was given, and the infusion rate was increased by 5 μg/kg/h. The maximum infusion rate was 40 μg/kg/h. The dose for neonates was halved. Propofol could also be administered as bridging sedative therapy up to the first 30 min following the IMP administration. A ketamine bolus was allowed when needed for procedures. Patients who underwent major surgery under anesthesia prior to the IMP treatment were carefully reviewed. Time, date, and duration of surgery, as well as every co‐medication given before and during the IMP treatment, were documented.

Pain and sedation were assessed using the COMFORT‐B [12], NISS, and NRS [13] scores every 3 h. Additional assessments were done 30 min following an adjustment of IMP and morphine administration. The pain scores always took precedence over the sedation scores to address patient distress. Other variables that were monitored included vital signs, fluid balance, severity of illness, blood gas, hematology, and clinical chemistry (liver and kidney functions).

Two blood samples for clonidine and midazolam assay were mandatory: One following the first loading dose and the second just before the end of the IMP treatment. Additional blood samples were taken during routine clinical procedures with a maximum number of six samples per patient. A baseline sample drug assay was taken before the IMP treatment from patients who received clonidine or midazolam within 5 days preceding the screening to determine subject eligibility for enrollment. The PK samples were collected using arterial or venous catheters and puncture. The sample volume was within 0.6 mL to 1.0 mL. Plasma concentrations of clonidine, midazolam, morphine, and their main metabolites (1‐OH midazolam, morphine 3‐glucuronide [M3G], and morphine 6‐glucuronide [M6G]) were measured using a specially developed assay [14].

Genetic variants from genes coding for metabolism enzymes (CYP3A4, CYP3A5, CYP2D6, UGT2B7, POR, COMT, and MC1R) and specific receptors involved in the mechanism of clonidine, midazolam, and morphine (BCB1, GABA, MDR1, MRP1, MRP2, MRP4, BRCP, ADRA2A, OPRM1, OCT1, ABCC3, IL‐1Ra, IL‐1b, ARRB2, and STAT6) were assessed.

### 2.3 Pharmacometric Modeling

Separate pharmacokinetic (PK) models were developed for clonidine and midazolam. One‐ and two‐compartment models were tested to define the basic structural PK model of both drugs. An additional compartment for 1‐OH midazolam was tested in the midazolam model. It was assumed that midazolam was entirely metabolized to 1‐OH midazolam.

In both PK models, body weight was included using allometric scaling (0.75 exponent for clearances and 1 for volumes), and a postmenstrual age (PMA)‐based sigmoidal maturation function was used to describe the influence of age on the clearances of clonidine, midazolam, and 1‐OH midazolam. Both body weight and age were included a priori in the PK models [15]. The parameters of the maturation function were fixed to values published by Larsson et al. [16] for clonidine and Anderson et al. [17] for midazolam.

Variables describing the kidney function (creatinine) and liver function (aspartate aminotransferase, alanine aminotransferase, gamma‐glutamyl transferase, and bilirubin) were tested as covariates for parameters in both models using an exponential function.

A two‐step process was used to evaluate the influence of selected single nucleotide polymorphisms (SNPs) on the PK parameters of clonidine and midazolam. First, a screening process was conducted to increase the power of the analysis and avoid false positives [18]. The screening consisted of testing the genetic association between variants and individual clearances using multiple linear regression. An additive genetic model was used, and a Bonferroni correction with a significance level of 0.2 was applied. The SNPs selected by the screening were then tested in the nonlinear mixed effect PK model as covariates on the clearance. The heterozygous and homozygous mutant types were grouped into one category. The SNPs with a minor allelic frequency below 5% were excluded from the analysis, and the remaining SNPs were tested for Hardy–Weinberg equilibrium. Genetic associations with PKPD parameters were not formally tested due to sample size and study design limitations.

### 2.4 Pharmacokinetic–Pharmacodynamic Modeling

PKPD models were developed to establish a relationship between drug concentration and effect assessed using the COMFORT‐B score (range 6–31). Individual predicted concentrations of clonidine and midazolam were used as drivers of the sedative effect in the analysis. The effect of 1‐OH midazolam PK on the score was not explored due to its small effect in comparison to midazolam. Separate models for clonidine and midazolam were first built, and then a joint model using observed data of both drugs was developed. A continuous inhibitory sigmoid Emax model was first tested. Then, the COMFORT‐B scores were treated as categorical, and a proportional odds model as well as a bounded integer model were tested [19].

The inclusion of a postanesthesia effect in the Emax model for the patients who underwent major surgery with anesthesia before starting the IMP treatment was tested as described in the PKPD model published by Peeters et al. which was developed to optimize the dose of midazolam in nonventilated infants after major surgery [20]. The postanesthesia effect was assumed to wash out over time following the surgery using an Emax model, resulting in a more awake sedation level to a maximum estimated score (SMAX) for sedation. The postanesthesia effect (PA) was implemented using an Emax model as follows:

$$\begin{array}{c} \mathrm{PA}=\text{BASE}+\text{PAEFF}\\ \text{PAEFF}=\frac{\text{PAEMAX}\cdot \mathrm{TPS}}{\mathrm{TPS}50+\mathrm{TPS}}\\ \text{PAEMAX}=\text{SMAX}-\text{BASE} \end{array}$$

where BASE is the score at the end of the surgery and PAEFF is the postanesthesia effect; SMAX corresponds to the maximal score obtained once the effect of the anesthetic used for the surgery is gone, PAEMAX is the maximal postanesthesia effect from BASE (difference between BASE and SMAX); TPS is the time after surgery in hours and TPS50 is the time after surgery at half maximum postanesthesia effect in hours.

To reflect the complete anesthesia induced during the surgery, the BASE parameter was fixed to 6 (minimal COMFORT‐B score). The maximum drug effect (Emax) was fixed to 6, as it was done in the model developed by Peeters et al. [20] to increase the model stability.

The effect of comedications (morphine, propofol, and ketamine) on the COMFORT‐B score was also tested one by one using an additive model. A PK model for morphine was developed using the collected observed morphine concentrations, and the effect of morphine was implemented using an Emax model driven by the individual predicted concentrations [S9]. Unlike morphine, ketamine and propofol concentrations were not collected; therefore, a K‐PD Emax model was used.

The total effect on the COMFORT‐B score (EFFECT) was characterized as follows:

‐ For the patients who underwent surgery:

EFFECT=PA−IMPEFF−CMEFF

‐ For the patients who did not undergo surgery:

EFFECT=B0−IMPEFF−CMEFF

where PA is the postanesthesia effect after surgery, IMPEFF is the IMP effect and CMEFF is the co‐medication effect. B0 corresponds to the baseline score which was estimated or fixed.

The common parameter for both clonidine and midazolam in the joint Emax model was the time after surgery at half maximum postanesthesia effect. Those parameters that could not be estimated by the joint model were fixed to the values estimated by the separate models. The effect of clonidine and midazolam on the scores was combined using an additive model as the patients were randomized to receive either clonidine or midazolam.

### 2.5 Model Evaluation and Software

The models were evaluated using visual predictive checks (VPC). Goodness‐of‐fit plots included plots of observations versus population and individual predictions, conditional weighted residuals (CWRES) versus time, and individual profile plots. Bootstrap analyses were used to investigate the parameter stability. Modeling was undertaken using NONMEM version 7.4 (ICON Development Solutions, USA) using the first‐order conditional estimation algorithm with interaction (FOCEI). The VPC and bootstrap were generated using Perl‐speaks NONMEM (PsN). Plots and data management were performed using R version 3.2.

### 2.6 Simulations

Simulations using the final PK and PKPD models were performed with doses of clonidine and midazolam used clinically. A database of 1000 patients with different demographic characteristics (PMA, weight, and sex) was generated using the Sumpter function to take into account the change of weight with PMA [21]. The simulations were limited to a 12‐h duration. A baseline score of 15 was used to simulate the COMFORT‐B score.

## 3 Results

### 3.1 Patients and Demographics

There were only 28 patients included in the study before premature termination. Thirteen received midazolam and 15 received clonidine. Morphine was administered to all the patients as background for pain. In addition, 10 children from the clonidine arm and 6 from the midazolam arm received propofol as a bridging sedative drug. Thirty‐six and 37 assay samples were used as PK data observations to build the clonidine and midazolam PK models, respectively. Two to four samples of clonidine or midazolam were available for each patient. Baseline samples with concentrations above the limit of quantification from one patient in the clonidine arm and four in the midazolam arm were available. The percentage of values below the limit of quantification for both drugs was below 5%. All 28 patients were included in the PKPD models. A total of 317 and 306 observed COMFORT‐B scores were used to build the midazolam and clonidine PKPD models, respectively. The demographic characteristics of the patients as well as the COMFORT‐B score data used as PD endpoints are summarized in Table 1.

**TABLE 1:** Table summarizing the demographic characteristics of the patients included in the analysis and the COMFORT‐B data by patient.

	Clonidine median (range)	Midazolam median (range)
Body weight (kg)	4.0 (2.7–16.1)	3.3 (2.0–16.7)
Gestational age (weeks)	40 (36.3–40)	40 (36.3–40)
Postnatal age (days)	41 (0–1419)	4 (0–2023)
Postmenstrual age (weeks)	45.9 (36.7–242.7)	40.1 (36.3–371.9)
Sex (M/F)	9/6	5/8
Surgery (yes/no)	7/8	11/2
Bridging propofol (yes/no)	10/5	6/7
COMFORT‐B score	13 (6–27)	12 (6–26)
Number of score data point	25 (9–47)	23 (18–38)
Assessment time (h)	62.5 (13.5–107.3)	62.7 (33.2–91.6)

**FIGURE 1:**
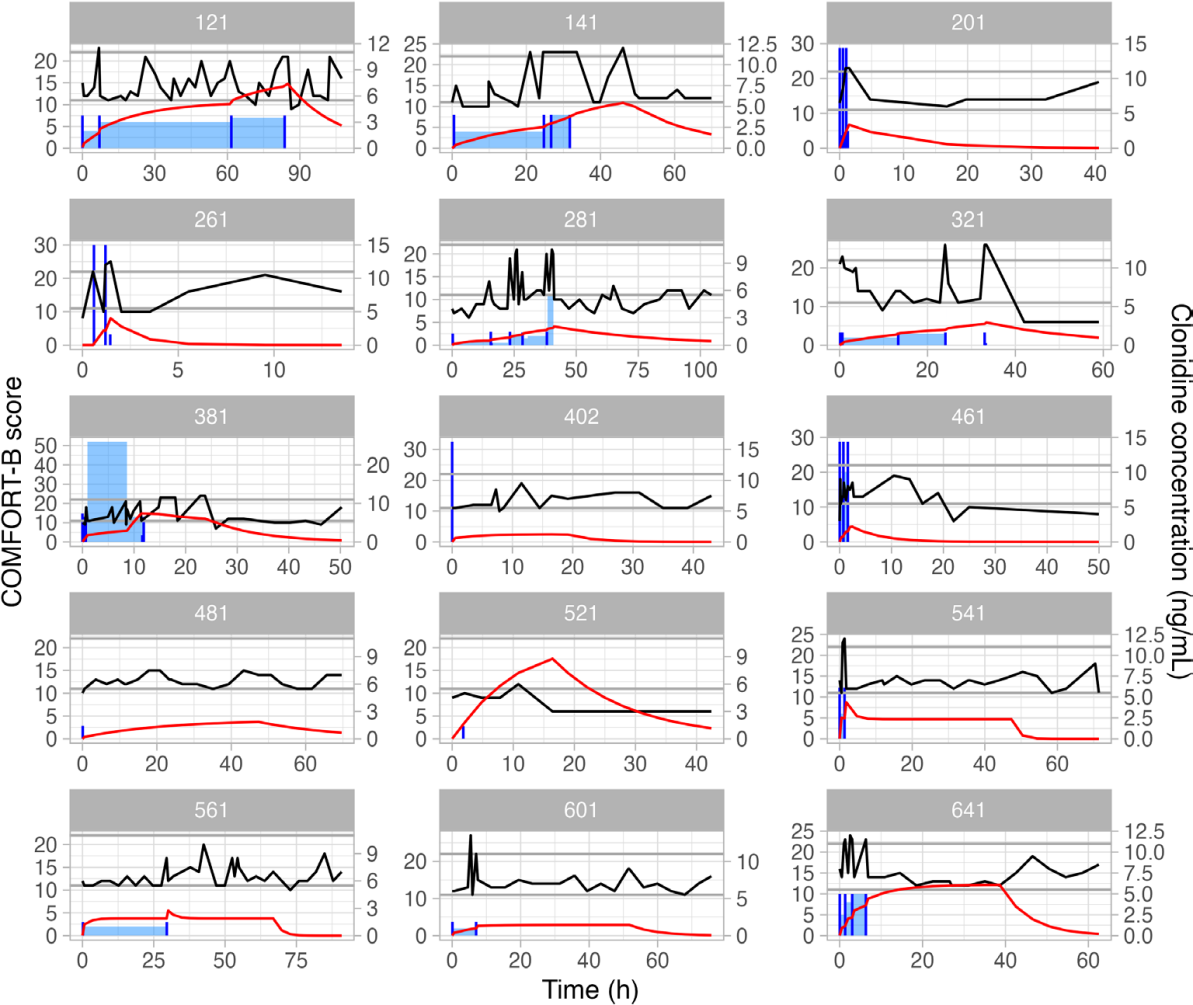
Individual plots of the patients receiving clonidine showing drug concentration profile (red line), COMFORT‐B score profile (black line), clonidine loading dose (vertical dark blue line), and clonidine infusion rate (blue shaded area).

**FIGURE 2:**
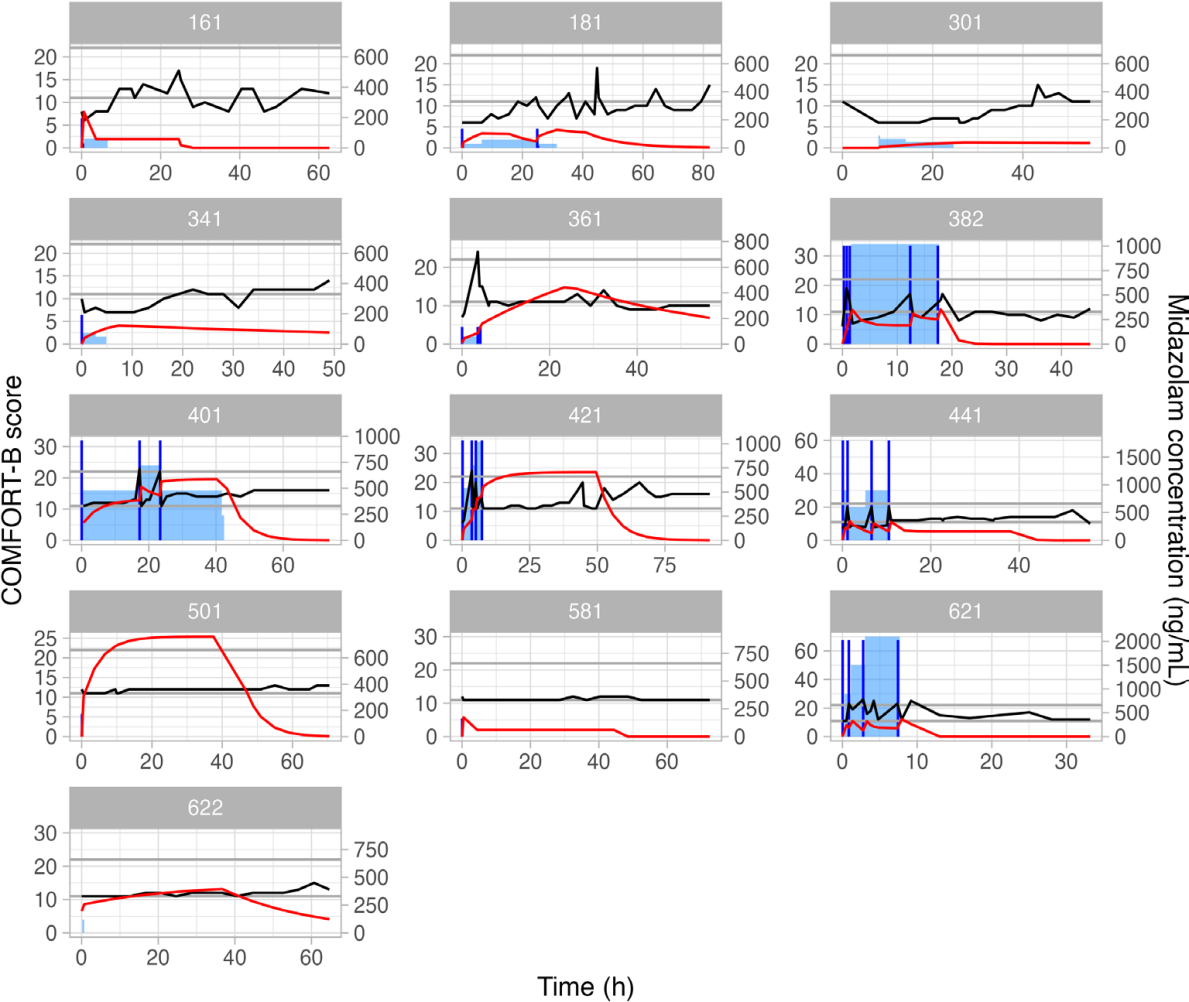
Individual plots of the patients receiving midazolam showing drug concentration profile (red line), COMFORT‐B score profile (black line), midazolam loading dose (vertical dark blue line), and midazolam infusion rate (blue shaded area).

Individual plots show drug concentration, COMFORT‐B score, IMP dose for clonidine (Figure 1) and midazolam (Figure 2).

### 3.2 Pharmacometric Modeling

The clonidine PK data were best described by a one‐compartment model, and the model that best fitted the midazolam PK data was a one‐compartment model for midazolam with an additional metabolite compartment for 1‐OH midazolam [S3, S4]. It was assumed in the model that midazolam was metabolized entirely to 1‐OH‐midazolam. The PK data below the limit of quantification were included in the analysis by dividing the respective limit of quantification for clonidine and midazolam by 2.

The parameter estimates from the clonidine and midazolam PK models are presented in Table 2. No other covariate had a significant effect on the PK parameters of clonidine and midazolam. In addition, the screening process was not able to identify any significant association between genetic variants and individual clearances for both drugs.

**TABLE 2:** Estimates from the final clonidine and midazolam PK models.

Drug	Parameter	Estimate (RSE)	Bootstrap median (90% CI)
Clonidine	CL_clon_ (L/h/70 kg)	28.0 (20)	28.1 (19.2–37.1)
	V_clon_ (L/70 kg)	202.4 (24)	202.2 (122.8–289.3)
	IIV CL_clon_ (%)	49.6 (47)	46.9 (20.7–65.6)
	IIV V_clon_ (%)	87.7 (32)	85.0 (57.4–106.8)
	Err prop_clon_ (%)	43.6 (7)	43.6 (20.7–56.6)
	PMA_50_clon_	61.6 FIX	—
	Hill_clon_	2.42 FIX	—
Midazolam	V_mid_ (L/70 kg)	85.8 (30)	75.6 (37.7–124.5)
	CLm_mid_ (L/h/70 kg)	33.4 (32)	35.3 (22.3–61.7)
	Vm_mid_ (L/70 kg)	90.8 (67)	87.3 (39.0–222.9)
	CLom_mid_ (L/h/70 kg)	211.6 (12)	214.7 (178.5–295.5)
	IIV CLm_mid_ (%)	91.7 (41)	84.9 (42.4–113.1)
	IIV V_mid_ (%)	133.4 (54)	131.1 (66.3–236.2)
	Err prop_mid_ (%)	46.9 (39)	45.8 (22.4–58.3)
	Err add_mid_ (ng/mL)	1.24 (69)	1.25 (0.24–1.79)
	Err propm_mid_(%)	57.4 (31)	57.4 (42.4–72.8)
	Err addm_mid_ (ng/mL)	0.023 (23)	0.023 (0.014–0.037)
	PMA_50_mid_	73.6 FIX	
	Hill_mid_	3 FIX	

*Note:* CL_clon_ and V_clon_ are the clonidine clearance and volume of distribution, respectively. For midazolam, V_mid_ is the volume of distribution, CLm_mid_ is the metabolite formation clearance, Vm_mid_ is the volume of the metabolic compartment, and CLom_mid_ is the clearance out of the metabolic compartment. PMA_50_clon_ and PMA_50_mid_ are the PMA (weeks) when the maturation has reach 50%, and Hill_clon_ and Hill_mid_ are the shape parameter for clonidine and midazolam, respectively. RSE is the relative standard error (from NONMEM covariance step), IIV is the interindividual variability. FIX means that the value of the parameter was fixed a priori in the model. Err prop and Err add correspond to the error proportional and additive, respectively. CI is the confidence interval.

### 3.3 Pharmacometric–Pharmacodynamic Modeling

Separate models for clonidine and midazolam were first developed. The model that best fitted the data for both drugs was a sigmoid inhibitory Emax model [S5]. For both models, the inclusion of a postanesthesia effect for the patients who underwent major surgery provided an improvement in fit (midazolam: Δ OFV = 28.1 *p* <10^−5^ and clonidine: Δ OFV = 361 *p* <10^−5^). In the midazolam model, the baseline scores were set to the individual observed values whereas the baseline was a parameter estimated in the clonidine model.

An effect of propofol included using an Emax K‐PD model was found on the COMFORT‐B score (Δ OFV = 21.1 *p* = 2.10^−5^) for the midazolam arm only. The propofol maximal effect and EC50 were fixed to published values [22]. No significant effect of other co‐medication including morphine was found.

Data from both the midazolam and clonidine groups were combined into a joint sigmoid inhibitory Emax model with the midazolam parameter estimates fixed to the values estimated by the separate midazolam model [S6]. The parameter estimates from the joint model are presented in Table 3. The model was well evaluated by the prediction‐corrected VPCs [S7, S8].

**TABLE 3:** Estimates from the final joint PKPD model.

Drug	Parameter	Estimate (RSE)	Bootstrap median (90% CI)
Midazolam	EC_50_ (ng/mL)	186.0 (−)	—
	PAEMAX	9.3 (−)	—
	IIV EC_50_ (%)	246.6 (−)	—
	Err prop (%)	24.9 (−)	—
Both drugs	TPS50 (h)	0.11 (68)	0.13 (0.03–0.36)
	BASE	6 FIX	—
	Emax	6 FIX	—
Clonidine	EC_50_ (ng/mL)	2.73 (9)	5.23 (0.41–31.8)
	PAEMAX	11.8 FIX	—
	B0	15.6 (4)	15.6 (14.3–16.6)
	IIV EC_50_ (%)	525 (52)	473 (225–1355)
	Err prop (%)	28.2 (14)	28.1 (23.6–31.6)

*Note:* BASE is the score at the end of the surgery, PAEMAX is the maximal postanesthesia effect from BASE, and TPS50 is the time postsurgery at half maximum postanesthesia effect in hours. Emax is the maximum drug effect, EC50 is the concentration to reach 50% of the maximum drug effect. RSE is the relative standard error (from NONMEM covariance step), IIV is the interindividual variability. FIX means that the value of the parameter was fixed a priori in the model. Err prop corresponds to the proportional error. CI is the confidence interval.

### 3.4 Simulations

**FIGURE 3:**
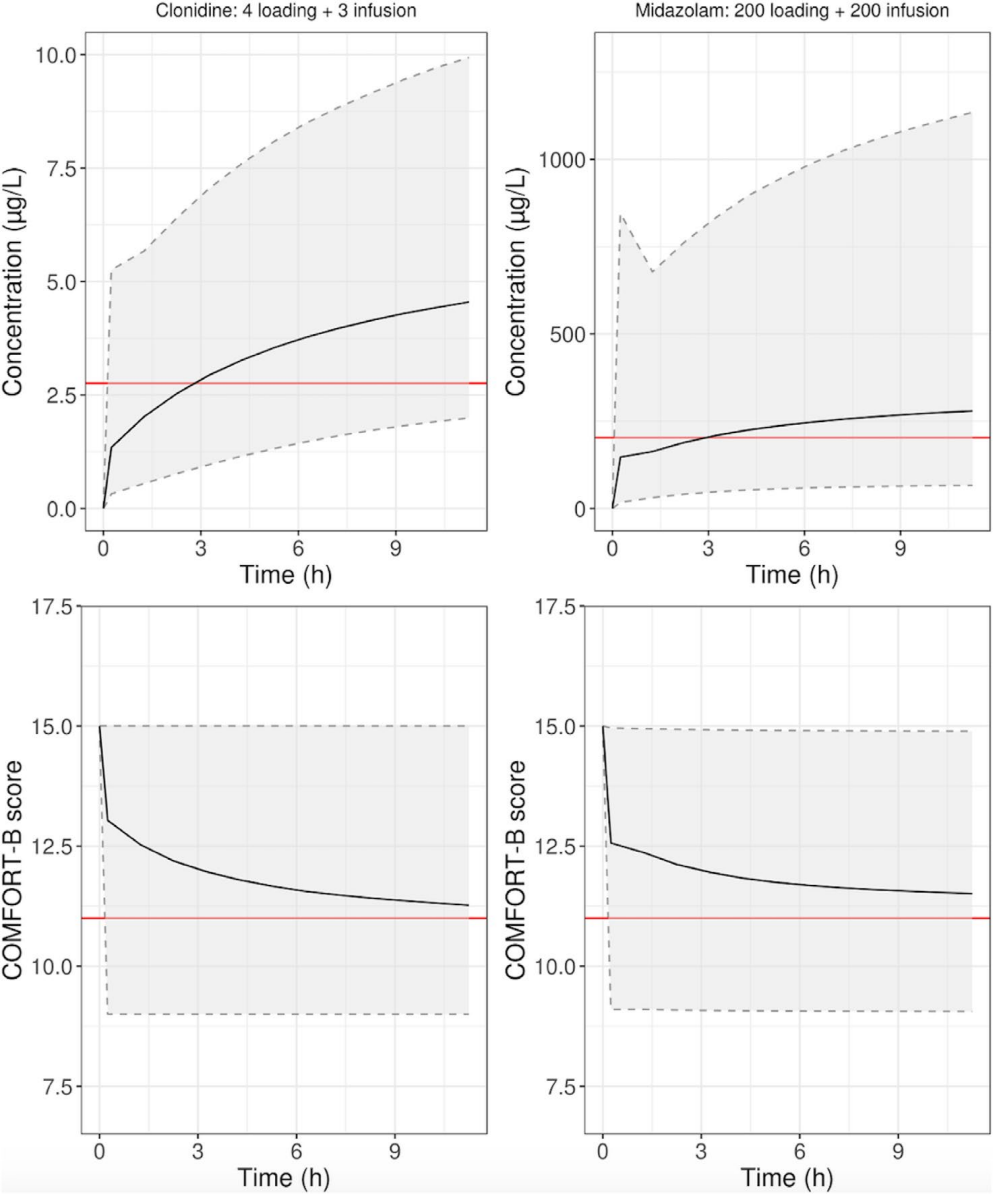
Both graphs on the top present the simulated concentration of midazolam (right) and clonidine (left) after administration in children older than 28 days of 200 μg/kg followed by an infusion of 200 μg/kg/h for midazolam and 4 μg/kg/h followed by an infusion of 3 μg/kg/h for clonidine. The red line represents the EC50 defined using the PKPD model. The graphs on the bottom show the change in COMFORT‐B score following the simulated concentration of clonidine (left) and midazolam (right) produced using the final PKPD model. The red line is the minimal score for which the level of sedation is considered adequate.

Midazolam concentrations and COMFORT‐B scores were simulated for a loading dose of 200 μg/kg followed by 200 μg/kg/h in children older than 28 days (Figure 3). The clonidine simulations for a dose of 4 μg/kg followed by 3 μg/kg/h are also shown in Figure 3. For both drugs, the estimated median reaches the EC_50_ estimated by the PKPD model 3 h following the first dose administration. A high variability in terms of concentration and COMFORT‐B score is predicted for both drugs.

## 4 Discussion

The CloSed study [2] was a double‐blind, multicenter, phase III randomized controlled trial that was prematurely terminated. Data from the study were used to develop PK and PKPD models for clonidine and midazolam to inform on the optimal use of both drugs in mechanically ventilated children. Data from published PK analyses (priors) were used to augment the analysis. A joint inhibitory sigmoid model including a postanesthesia effect for the patients who underwent major surgery before treatment was successfully built to describe the concentration–effect relationship of clonidine and midazolam using the COMFORT‐B score. The simulations showed that the doses commonly used in clinical practice of both sedatives should be increased to provide an optimal sedation management.

The clearances estimated by the PK models are consistent with those reported in the literature for clonidine (14–45 L/h/70 kg) [23] and midazolam (12–58 L/h/70 kg) [17]. Potts et al. reported an elimination clearance for clonidine [8] of 14.6 L/h/70 kg (CV 35.1%), Larsson et al. [16] 18 L/h/70 kg (CV 30%), whereas our study estimated 28 L/h/70 kg (RSE 20%). These differences may be explained by variations in patient populations (our study included critically ill children in the PICU who may have altered pharmacokinetics) and methodological factors such as sampling. Midazolam clearance reported by Peeters et al. [20] (0.157 L/min in infants with median weight 9.4 kg) differs in units and scaling from our allometrically scaled estimate normalized to 70 kg. Once differences in body size, age, and clinical context are accounted for, our results align well with the literature. These observations underscore the importance of population‐specific parameter estimation for accurate dosing guidance. A PMA‐based sigmoidal maturation function and an allometric weight scaling were used to describe the clearance of both drugs, allowing extrapolation to different populations.

The PKPD model developed by Peeters et al. [20] to inform on the optimal dose of midazolam for sedation of nonventilated infants after major surgery using COMFORT‐B score was used as a reference to build the joint PKPD model for clonidine and midazolam in our study. Peeters et al. also included a postanesthesia effect and an Emax model with a maximal effect fixed to the lowest possible score of 6. One of the main differences was the baseline score during surgery that was fixed in our model to the minimal COMFORT‐B score. The EC_50_ for midazolam estimated by our model (186 ng/mL) is similar to the one estimated by the model developed by Peeters et al. (200 ng/mL), which also used the COMFORT‐B score as an efficacy measure. A study conducted by Tolia et al. [24] suggested that a mean peak concentration of midazolam around 200 ng/mL provided adequate sedation in children undergoing endoscopy. When using EEG as a measurement for hypnosis, Mandema et al. [25] estimated an EC_50_ of approximately 77 ng/mL in healthy adult patients. The difference in EC_50_ can be explained by the difference in procedure type and PD tool used in their study. The EC_50_ defined for clonidine and midazolam by our model was used as a target concentration in the simulation performed to inform on the optimal dosing regimens of both drugs.

The simulations performed using the final PKPD model show that a loading dose of clonidine higher than the loading dose of 2 and 3 μg/kg currently prescribed in clinical practice should be administered to provide an adequate sedation during the first hours after administration. This finding is consistent with other reports that suggest a higher dose requirement for children nursed in an intensive care unit [9]. However, an increased dose of both midazolam and clonidine is associated with increased adverse effects. It has been shown that clonidine loading doses and infusions up to 2 μg/kg are generally well tolerated in the PICU; however, higher doses were associated with adverse effects, notably bradycardia and hypotension [9]. Such adverse effects could be better controlled by using target‐controlled infusion, as is done for other analgesics and sedatives. There remains a paucity of concentration–adverse effect relationships available for these two drugs that can be used by clinicians to best determine loading or maintenance dose.

Recently, it has been shown that bias in the estimated dose/exposure relationship can occur when analyzing data from dose titration trials such as the CloSed study [26]. This bias is caused by patients receiving high doses but having poor responses and patients receiving low doses but having good responses. Using individual predicted concentrations instead of dose as the driver of the effect, as it was done in this PKPD analysis, limits the risk of such bias.

The main limitation of the analysis was the small sample size that impacted the result of the analysis. Firstly, it was not feasible to prove noninferiority of clonidine compared to midazolam using logistic regression. Secondly, the maturation function parameters for the PK models were fixed to published values [16, 17] but since the effect of ontogeny on clonidine and midazolam clearance has been well described in the literature, the fit was not worsened. In addition, the PKPD models estimated a large interindividual variability on the EC_50_ and were not able to estimate the Emax nor the baseline B0 for clonidine. Finally, some parameters such as TPS50 were estimated with lower precision (RSE > 50%). Despite these limitations, the PK and PKPD models developed in this analysis adequately described the observed data and provided a useful framework for performing simulations to inform optimal dosing of clonidine and midazolam. It is important to note that a high degree of variability was observed in both predicted concentrations and sedation responses, reflecting the complex and heterogeneous nature of the critically ill pediatric population. This variability underscores the challenges in optimizing sedation regimens but also highlights the value of model‐based simulations to explore dosing strategies across diverse patient profiles. While variability may limit the precision of individual predictions, the simulations provide a valuable framework for guiding dose selection and adjusting regimens to improve overall sedation management in this vulnerable group.

Numerous adverse events (nonrelated to clonidine or midazolam) were experienced by most patients, and co‐medications were frequently needed in both arms. Therefore, the COMFORT‐B score might be impacted by additional factors other than the drugs investigated.

The lack of effect of morphine and its metabolites on the COMFORT‐B sedation score may be explained by several factors. First, all patients received a continuous morphine infusion, which likely led to stable plasma concentrations and minimal between‐individual variability, limiting the model's ability to detect an additive effect on sedation. Second, the COMFORT‐B scale is primarily designed to assess sedation and behavioral distress, not analgesia, and may therefore be suboptimal for capturing morphine's effects. Third, the absence of a measurable impact could also reflect morphine tolerance or subtherapeutic exposure levels in this specific cohort. Finally, because clonidine or midazolam was always coadministered, isolating morphine's contribution to the sedation score was inherently difficult.

Although genetic variability in drug‐metabolizing enzymes and receptor targets may influence the pharmacokinetics and pharmacodynamics of clonidine and midazolam, our exploratory pharmacogenetic analysis did not identify any statistically significant associations between the selected SNPs and individual clearance estimates. This finding is likely due to the small sample size, which limits statistical power and the ability to detect modest or rare variant effects. Additionally, given the limited data and complexity introduced by multiple concomitant medications and sparse sampling, we did not extend the genetic analysis to pharmacodynamic outcomes. These limitations should be taken into account when interpreting our findings. Nonetheless, we believe it is important to report the lack of associations transparently, and the methodology used may still be informative for future studies. Larger, adequately powered studies are warranted to better evaluate the role of pharmacogenetic variability in this patient population.

Even though the CloSed trial was prematurely terminated, the data collected during the study could be used to perform PKPD modeling and simulations that improve our understanding of the optimal dose of clonidine and midazolam in children by showing that the dose of both drugs should be increased to provide an adequate pain and sedation.

## 5 Conclusion

The modeling work done in this study improves our knowledge on the optimal use of clonidine and midazolam in the PICU. Population models were developed to characterize the relationship between drug concentrations and analgesic/sedative effect. The simulations performed show that to provide adequate sedation, clonidine doses should be increased up to 4 μg/kg loading dose followed by 3 μg/kg/h and midazolam dosed as 200 μg/kg loading dose followed by 200 μg/kg/h. These findings could be used in clinical practice to improve pain and sedation management in children.

## Conflicts of Interest

The authors declare no conflicts of interest.

## Supporting information

[S1] Primary Endpoint Analysis.
[S2] Dosing_Algorithm.
[S3] Diagnostic plots clonidine PK model.
[S4] Diagnostic plots midazolam PK model.
[S5] PKPD observed data.
[S6] Parameters estimated using the separate PKPD models.
[S7] Nonmem output PKPD model.
[S8] Diagnostic plots for final joint PKPD model.
[S9] Result PK model morphine.

## Data Availability

The data that support the findings of this study are available on request from the corresponding author. The data are not publicly available due to privacy or ethical restrictions.
