# Supplementary material for: Pharmacokinetic and Pharmacodynamic Modeling of Clonidine and Midazolam for Sedation in Pediatric Intensive Care
Source: Paediatr Anaesth. 2025 Oct 4;35(12):1053–62. doi: 10.1111/pan.70050 (PMC12603884; doi:10.1111/pan.70050)
Supplement: Supplementary file 1 — [S1] Primary Endpoint Analysis. [S2] Dosing_Algorithm. [S3] Diagnostic plots clonidine PK model. [S4] Diagnostic plots midazolam PK model. [S5] PKPD observed data. [S6] Parameters estimated using the separate PKPD models. [S7] Nonmem output PKPD model. [S8] Diagnostic plots for final joint PKPD model. [S9] Result PK model morphine. [file PAN-35-1053-s001.zip › Diagnostic plots clonidine PK model.pdf]

Diagnostic plots for clonidine PK model

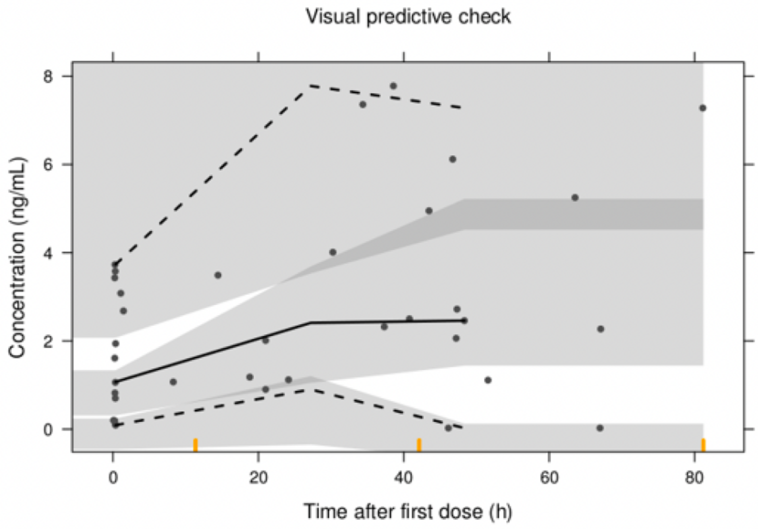

Figure 62: Visual Predictive Check produced using the parameters estimated by the final clonidine PK model. The shaded grey area is the 95 percent prediction interval. The black solid line is the median of the observed data; the black dashed lines are the 5 th and 95 th percentiles of the observed data.

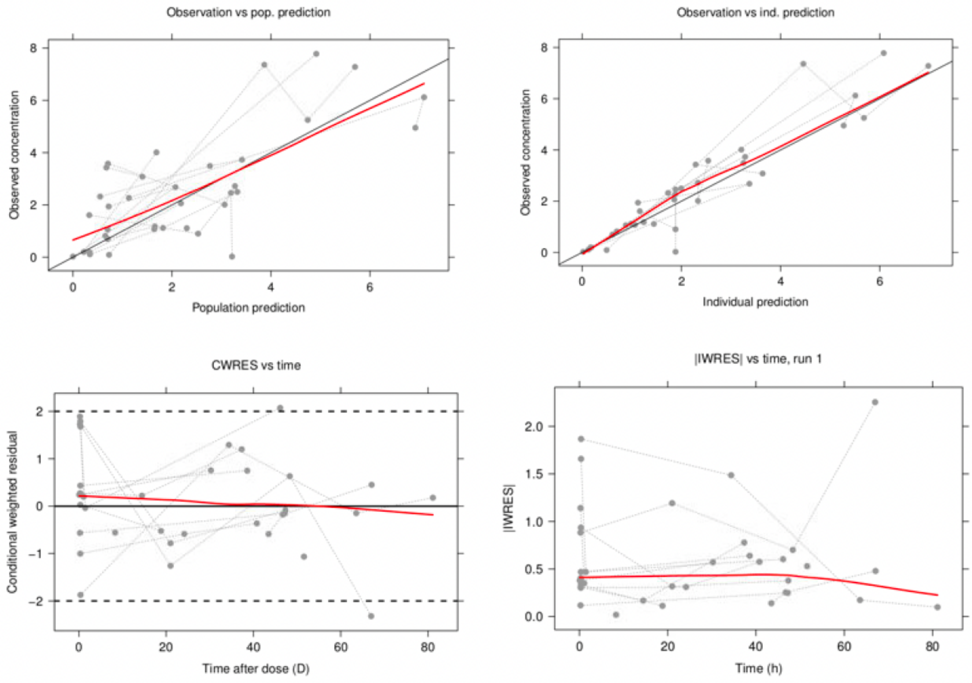

Figure 61: Goodness-of-fit plots of the final clonidine PK model. Plots of the observed concentration vs population predicted concentration (top left) and vs individual predicted concentration (top right), the CWRES versus time after dose (bottom left) and plot of the IWRES vs time after dose (bottom right) from the final fentanyl population PK model. The red line is the lowess line and the black line is the line of unity.
