# Supplementary material for: Pharmacokinetic and Pharmacodynamic Modeling of Clonidine and Midazolam for Sedation in Pediatric Intensive Care
Source: Paediatr Anaesth. 2025 Oct 4;35(12):1053–62. doi: 10.1111/pan.70050 (PMC12603884; doi:10.1111/pan.70050)
Supplement: Supplementary file 1 — [S1] Primary Endpoint Analysis. [S2] Dosing_Algorithm. [S3] Diagnostic plots clonidine PK model. [S4] Diagnostic plots midazolam PK model. [S5] PKPD observed data. [S6] Parameters estimated using the separate PKPD models. [S7] Nonmem output PKPD model. [S8] Diagnostic plots for final joint PKPD model. [S9] Result PK model morphine. [file PAN-35-1053-s001.zip › Diagnostic plots for final joint PKPD model.pdf]

Diagnostic plots for clonidine using final joint model

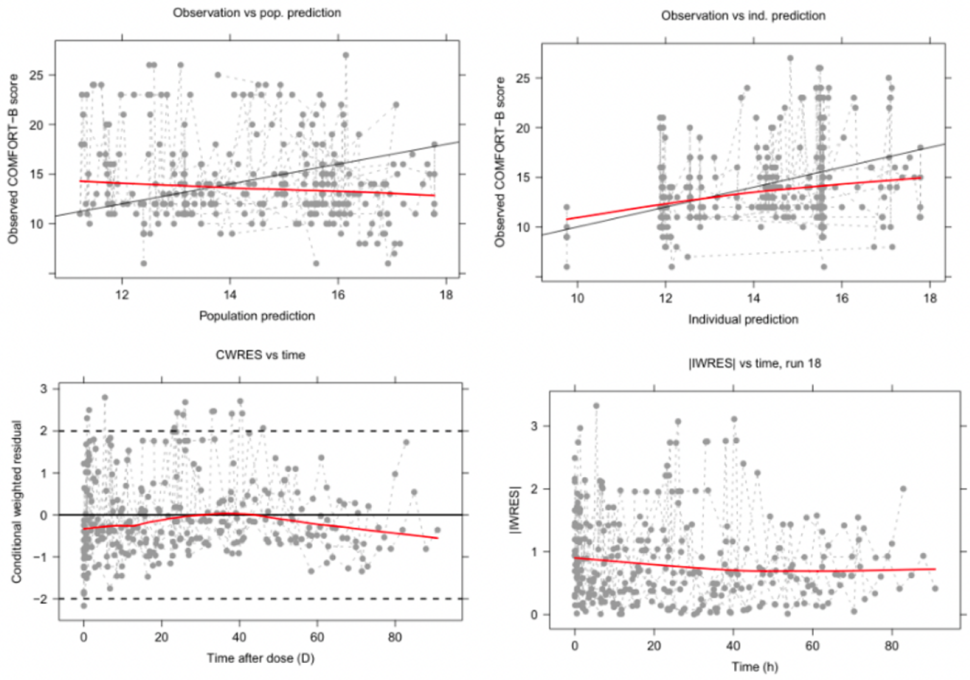

Figure 79: Goodness-of-fit plots of clonidine using the final joint PK/PD model. Plots of the observed score vs population predicted score (top left) and vs individual predicted score (top right), CWRES versus time after dose (bottom left) and plot of the IWRES vs time after dose (bottom right). The red line is the lowess line and the black line is the line of unity.

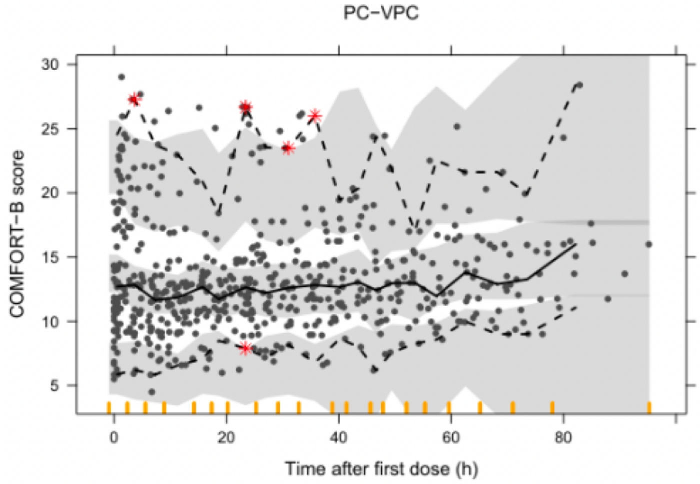

Figure 80: VPC of clonidine produced using the parameters estimated by the final joint PK/PD model. The shaded grey area is the 95 percent prediction interval. The black solid line is the median of the observed data; the black dashed lines are the 5 th and 95 th percentiles of the observed data.

Diagnostic plots for midazolam using final joint model

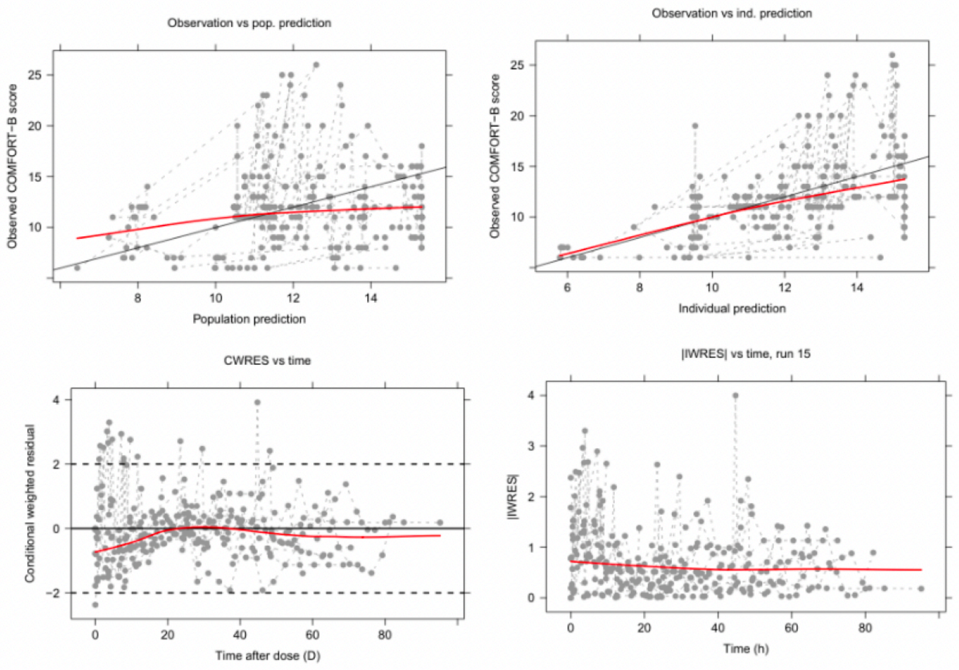

Figure 77: Goodness-of-fit plots of the final midazolam PK/PD model. Plots of the observed score vs population predicted score (top left) and vs individual predicted score (top right) CWRES versus time after dose (bottom left) and plot of the IWRES vs time after dose (bottom right). The red line is the lowess line and the black line is the line of unity.

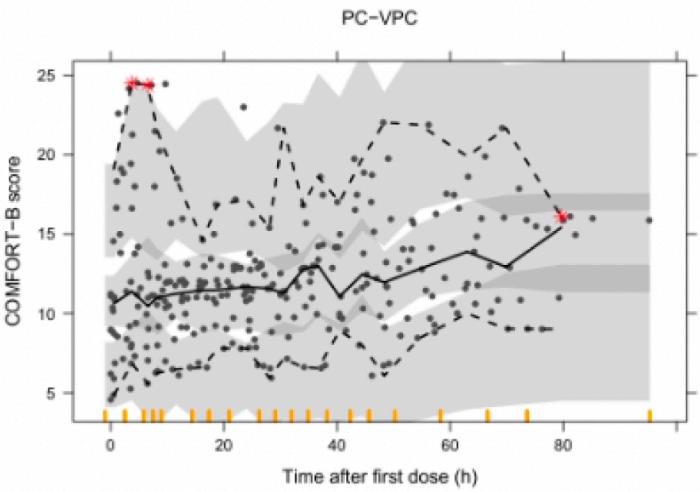

Figure 78: VPC produced using the parameters estimated by the final midazolam PK/PD model. The shaded grey area is the 95 percent prediction interval. The black solid line is the median of the observed data; the black dashed lines are the 5 th and 95 th percentiles of the observed data.
