# Supplementary material for: Pharmacokinetic and Pharmacodynamic Modeling of Clonidine and Midazolam for Sedation in Pediatric Intensive Care
Source: Paediatr Anaesth. 2025 Oct 4;35(12):1053–62. doi: 10.1111/pan.70050 (PMC12603884; doi:10.1111/pan.70050)
Supplement: Supplementary file 1 — [S1] Primary Endpoint Analysis. [S2] Dosing_Algorithm. [S3] Diagnostic plots clonidine PK model. [S4] Diagnostic plots midazolam PK model. [S5] PKPD observed data. [S6] Parameters estimated using the separate PKPD models. [S7] Nonmem output PKPD model. [S8] Diagnostic plots for final joint PKPD model. [S9] Result PK model morphine. [file PAN-35-1053-s001.zip › Nonmem output PKPD model.pdf]

```
$SUBROUTINE ADVAN9 TOL=9
$MODEL     COMP(1)
           COMP(2)
           COMP(3)
           COMP(4)


$PK


V = IV
CLTM = ICLTM
VM = IVM
CLOM = ICLOM
V1 = IV1
CL = ICL


TVEC50 = THETA(1)
EC50   = TVEC50 * EXP(ETA(1))

TVEMAX = THETA(2)
EMAX   = TVEMAX * EXP(ETA(2))

TVHILL   = THETA(3)
HILL     = TVHILL * EXP(ETA(3))

TVPAEMAX = THETA(4)
PAEMAX   = TVPAEMAX * EXP(ETA(4))

TVTPS50 = THETA(5)
TPS50   = TVTPS50 * EXP(ETA(5))

TVBASE = THETA(6)
BASE   = TVBASE * EXP(ETA(6))

TVKDE = THETA(7)
KDE   = TVKDE * EXP(ETA(7))

TVEC50C = THETA(8)
EC50C   = TVEC50C * EXP(ETA(8))

TVPAEMAXC = THETA(9)
PAEMAXC   = TVPAEMAXC * EXP(ETA(9))

TVB0 = THETA(10)
B0   = TVB0 * EXP(ETA(10))
```

```
S1=V
S2=VM
S4=V1

 A_0(1) = BSL*V
 A_0(2) = BSL2*VM
 A_0(4) = BSL*V1

 K12  = CLTM / V
 K20  = CLOM / VM
 K40  = CL / V1


$DES
 DADT(1)=  - K12*A(1)
 DADT(2)=  K12*A(1) - K20*A(2)
 DADT(3)=  - KDE*A(3)
 DADT(4)=  - K40*A(4)


$ERROR
CP=A(1)/V
CPC=A(4)/V1
IF (SURG.EQ.1.AND.FLAG.EQ.1) PNSP = BASE + (PAEMAX * TPS/ (TPS50 + TPS))
IF (SURG.EQ.1.AND.FLAG.EQ.2) PNSP = BASE + (PAEMAXC * TPS/ (TPS50 + TPS))
IF (SURG.EQ.0.AND.FLAG.EQ.1) PNSP = B2
IF (SURG.EQ.0.AND.FLAG.EQ.2) PNSP = B0
MEF =   (EMAX * CP**HILL / (EC50**HILL + CP**HILL))
CEF =   (EMAX * CPC**HILL / (EC50C**HILL + CPC**HILL))
EMAXP = 6
EC50P = 1.76


PEF= (EMAXP * (A(3))  / (EC50P + (A(3))))
IF (FLAG.EQ.1) IPRED = PNSP -  MEF - PEF
IF (FLAG.EQ.2) IPRED = PNSP -  CEF
PROP=SQRT(SIGMA(1,1))*IPRED
ADD=SQRT(SIGMA(2,2))
IF (FLAG.EQ.1) Y= IPRED*(1 + EPS(1)) + EPS(2)
IF (FLAG.EQ.2) Y= IPRED*(1 + EPS(3)) + EPS(4)
W = SQRT(PROP**2+ADD**2)

IRES = DV-IPRED
IWRES = IRES/W
```

```
$THETA (186 FIX) ;EC50
$THETA (6 FIX) ;EMAX
$THETA (1 FIX); HILL
$THETA (9.3 FIX)  ; TPMAX
$THETA (0,1)   ;TP50
$THETA (6 FIX)    ;BASE
$THETA (9.57 FIX)    ;KDE
$THETA (0,3) ;EC50
$THETA (11.8 FIX)  ; TPMAX
$THETA (0,15)  ; B0


$OMEGA 6.06 FIX
$OMEGA 0 FIX
$OMEGA 0 FIX
$OMEGA 0 FIX
$OMEGA 0 FIX
$OMEGA 0 FIX
$OMEGA 0 FIX
$OMEGA 0.1
$OMEGA 0 FIX
$OMEGA 0 FIX

$SIGMA 0.061 FIX
$SIGMA 0 FIX
$SIGMA 0.01
$SIGMA 0 FIX
```
