# Supplementary material for: Pharmacokinetic and Pharmacodynamic Modeling of Clonidine and Midazolam for Sedation in Pediatric Intensive Care
Source: Paediatr Anaesth. 2025 Oct 4;35(12):1053–62. doi: 10.1111/pan.70050 (PMC12603884; doi:10.1111/pan.70050)
Supplement: Supplementary file 1 — [S1] Primary Endpoint Analysis. [S2] Dosing_Algorithm. [S3] Diagnostic plots clonidine PK model. [S4] Diagnostic plots midazolam PK model. [S5] PKPD observed data. [S6] Parameters estimated using the separate PKPD models. [S7] Nonmem output PKPD model. [S8] Diagnostic plots for final joint PKPD model. [S9] Result PK model morphine. [file PAN-35-1053-s001.zip › Parameters estimated using the separate PKPD models.pdf]

Parameter estimated by the separate PKPD models for Clonidine and Midazolam

Drug	Parameter	Estimate (RSE)
Midazolam	EC50 (ng/mL)	186.0 (61)
	PAEMAX	9.3 (11)
	IIV EC50 (%)	246.6 (55)
	Err prop (%)	24.9 (15)
	TPS50 (h)	0.11 (43)
Both drugs	BASE	6 FIX
	Emax	6 FIX
Clonidine	EC50 (ng/mL)	2.73 (7)
	PAEMAX	11.8 FIX
	B0	15.6 (4)
	IIV EC50 (%)	525 (56)
	Err prop (%)	28 (26)
	TPS50 (h)	0.069 (228)

BASE is the score at the end of the surgery, PAEMAX is the maximal postanesthesia effect from BASE and TPS50 is the time post-surgery at half maximum postanesthesia effect in hours. EMAX is the maximal effect, EC50 is the concentration to reach 50% of the maximal effect and IIV is the interindividual variability
