# Supplementary material for: Pharmacokinetic and Pharmacodynamic Modeling of Clonidine and Midazolam for Sedation in Pediatric Intensive Care
Source: Paediatr Anaesth. 2025 Oct 4;35(12):1053–62. doi: 10.1111/pan.70050 (PMC12603884; doi:10.1111/pan.70050)
Supplement: Supplementary file 1 — [S1] Primary Endpoint Analysis. [S2] Dosing_Algorithm. [S3] Diagnostic plots clonidine PK model. [S4] Diagnostic plots midazolam PK model. [S5] PKPD observed data. [S6] Parameters estimated using the separate PKPD models. [S7] Nonmem output PKPD model. [S8] Diagnostic plots for final joint PKPD model. [S9] Result PK model morphine. [file PAN-35-1053-s001.zip › Primary Endpoint Analysis.pdf]

PRIMARY ENDPOINT

METHODS

The primary aim of the trial was to assess the non-inferiority of continuous intravenous clonidine compared to midazolam in mechanically ventilated children in the PICU. The primary endpoint was sedation success or failure.

Sedation failure was defined using pain and sedation scores as follows:

• NRS < 4 and COMFORT-B > 22

• NRS < 4, 11 <= COMFORT-B >= 22 and NISS = 1

The statistical analysis was done using logistic regression with treatment, center, and age groups (group 1: PNA<=27 days, group 2: 27<PNA<=723 days and group 3: PNA>=730 days) as covariate at a one-sided significance level of alpha corresponding to 2.5%.

The statistical hypotheses were defined as follows:

- H0: OR <= δOR (clonidine inferior to midazolam)

- H1: OR > δOR (clonidine non inferior to midazolam)

With

$$OR = \frac{pC \cdot (1 - pM)}{(1 - pC) \cdot pM}$$

Where OR is the odds ratio, and pC and pM are the probabilities of sedation success in the clonidine and midazolam group, respectively. δOR is the non-inferiority margin which was predefined as a value of 0.583.

RESULTS

In the clonidine group, 8 patients were considered as sedation success, 4 as sedation failure and 3 were not assessable. In the midazolam group, 11 patients had sedation success and 2 had sedation failure based on the primary endpoint criteria. Because of the small sample size, a quasi-complete separation was detected in the logistic regression model that had been envisaged for the primary endpoint. There results of this analysis were considered unreliable, and these are therefore not presented. Non-inferiority of continuous intravenous clonidine compared to midazolam was not proven.
