# Supplementary material for: Pharmacokinetic and Pharmacodynamic Modeling of Clonidine and Midazolam for Sedation in Pediatric Intensive Care
Source: Paediatr Anaesth. 2025 Oct 4;35(12):1053–62. doi: 10.1111/pan.70050 (PMC12603884; doi:10.1111/pan.70050)
Supplement: Supplementary file 1 — [S1] Primary Endpoint Analysis. [S2] Dosing_Algorithm. [S3] Diagnostic plots clonidine PK model. [S4] Diagnostic plots midazolam PK model. [S5] PKPD observed data. [S6] Parameters estimated using the separate PKPD models. [S7] Nonmem output PKPD model. [S8] Diagnostic plots for final joint PKPD model. [S9] Result PK model morphine. [file PAN-35-1053-s001.zip › Result PK model morphine.pdf]

In total, 65 samples were used to developed the morphine PK model. Each sample provided a concentration of morphine and the two main metabolites M3G and M6G. One patient was excluded of the PK analysis because a dose was missing. Hence, 27 children were included to build the final model.
The number of samples by patient was between 2 and 5. Three children had positive concentration of morphine and metabolites before starting the IMP treatment. These data were used in the model as baseline concentrations.

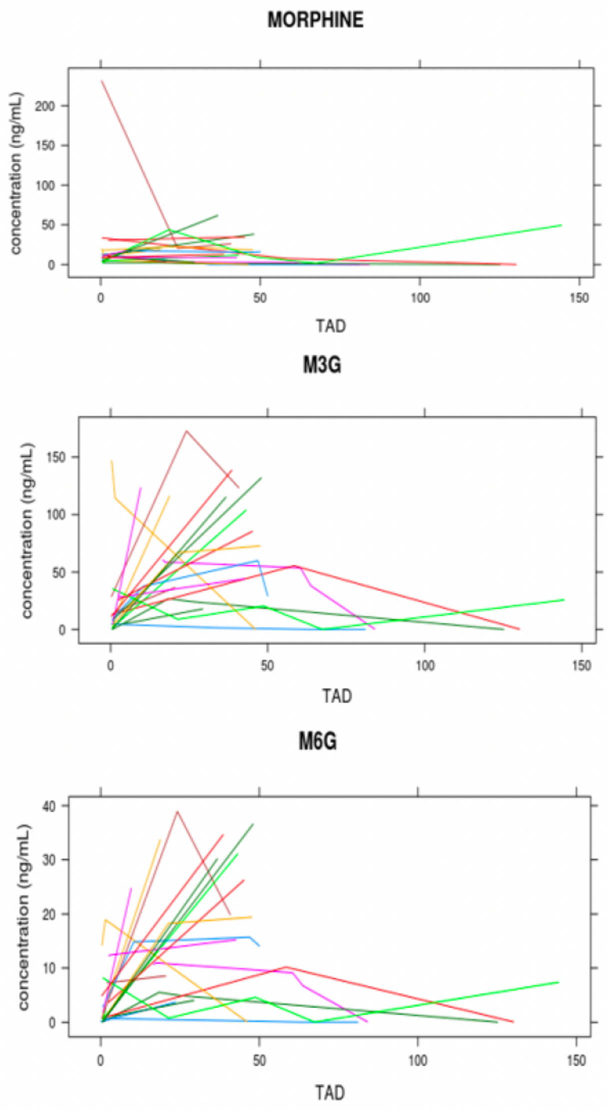

Observed concentration of morphine and its main metabolites M3G and M6G plotted against the time after dose administration (TAD). Each line represents a patient

The PK data were best described by a one-compartment model for morphine and one-compartment model for each metabolite (M3G and M6G). IIV was added on central volume of distribution (V1), formation clearance of M3G (CL3M) and M6G (CL6M), M3G volume of distribution (V3) and the metabolite clearance of M3G (CLom3). A combined error model for each compartment was used to describe the residual error.

The model was not able to estimate the morphine clearance (parameter estimated to non plausible values), therefore the model assumed that morphine was entirely metabolised to M3G and M6G.

Parameter	Estimate (RSE (%))	IIV (RSE (%))	Bootstrap median(5-95)
V1 (L/70kg)	103.6 (34)	116.6 (47)	110.3 (61.2 - 172.2)
CL3M (L/h/70kg)	81.8 (31)	117.4 (31)	83.8 (54.6 - 117.6)
V3 (L/70kg)	38.4 (26)	134.5 (46)	42.1 (24.3 - 67.8)
CLom3 (L/h/70kg)	16.3 (23)	99.4 (35)	15.7 (10.4 - 20.7)
CL6M (L/h/70kg)	6.5 (21)	48.9 (45)	6.8 (5.0 - 9.3)
V6 (L/70kg)	30 FIX (-)	-	-
CLom6 (L/h/70kg)	5.5 (23)	-	5.5 (3.9 - 9.6)
Crea CL3M	-1.3 (15)	-	-1.3 (-2.0 - 0.9)
Crea CLom6	2.0 (25)	-	1.9 (0.5 - 3.0)
Err prop parent (%)	54.7 (35)	-	52.0 (33.2 - 65.6)
Err add parent (ng/mL)	0.025 (6)	-	0.025 (0.024 - 0.025)
Err prop M3G (%)	54.7 (32)	-	50.9 (36.1 - 61.6)
Err add M3G (ng/mL)	0.30 (59)	-	0.24 (0.020 - 0.47)
Err prop M6G (%)	76.8 (90)	-	68.6 (34.6 - 183.3)
Err add M6G (ng/mL)	0.062 (60)	-	0.057 (0.018 - 0.092)

V1 is the central volume of distribution, CL3M and CL6M are the formation clearances of M3G and M6G, respectively. V3 and V6 correspond to the volume of the metabolic compartments. CLom3 and CLom6 are the clearance out of the metabolic compartment of M3G and M6G, respectively. Crea CL3M and Crea CLom6 are the index corresponding to the effect of creatinine level on CL3M and Clom6, respectively. RSE is

the relative standard error (from NONMEM covariance step), IIV is the interindividual variability. Err prop and Err add correspond to the error proportional and additive, respectively.

The estimation of the M6G volume of distribution induced an instability of the model leading an estimation to non plausible values for all parameters. Hence this parameter was fixed to a value published previously in a morphine model developed in children by Knibbe et al.
The parameters of the sigmoidal maturation function used to describe the formation clearances of both metabolites (CL3M and CL6M) were fixed to values published by Anand et al. Those used to estimate the metabolite clearances (CLom3 and CLom6) and central volume of distribution (V1) were fixed to values estimated by the morphine model developed by Bouwmeester et al.
In addition to weight and age, the model also found a significant influence of creatinine level on CL3M and CLom6.

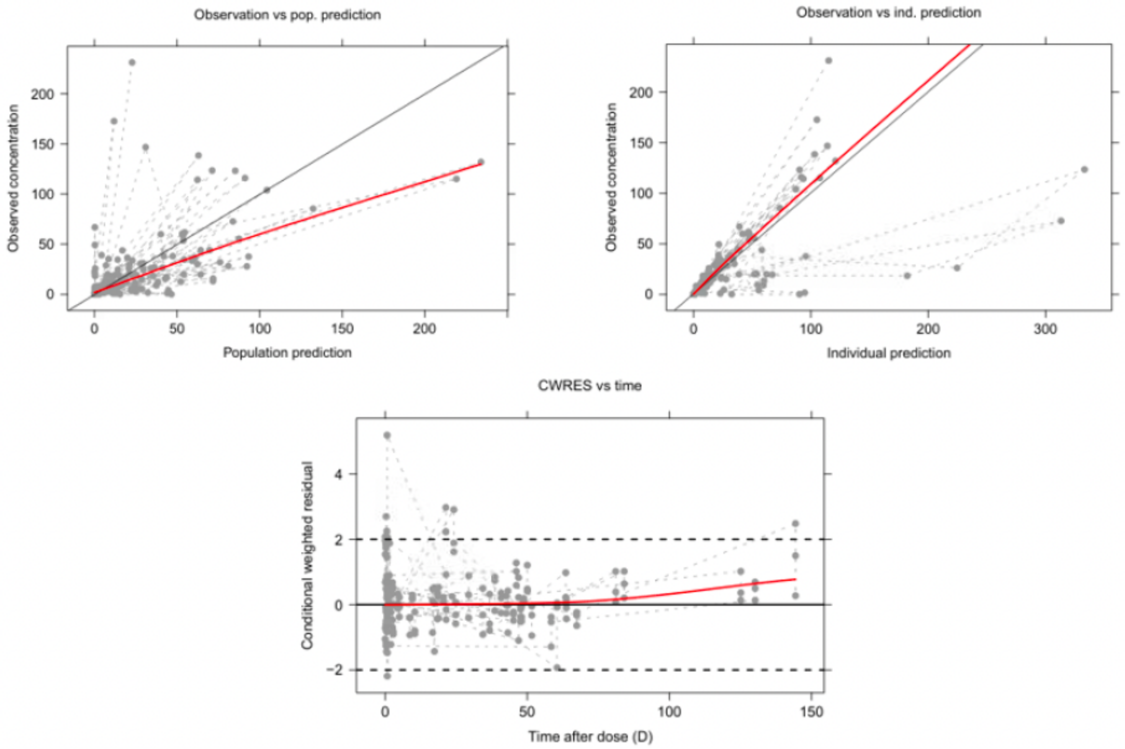

Goodness-of-fit plots of the final morphine PK model. Plots of the observed concentration vs population predicted concentration (top left) and vs individual predicted concentration (top right), the CWRES versus time after dose from the final morphine population PK model. The red line is the lowess line and the black line is the line of unity.

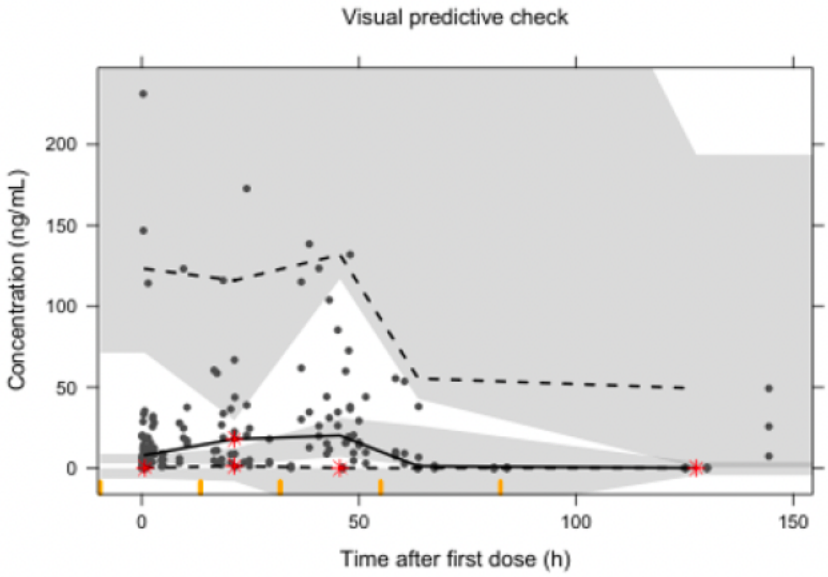

Visual Predictive Check produced using the parameters estimated by the final morphine PK model. The shaded grey area is the 95 percent prediction interval. The black solid line is the median of the observed data; the black dashed lines are the 5 th and 95 th percentiles of the observed data.

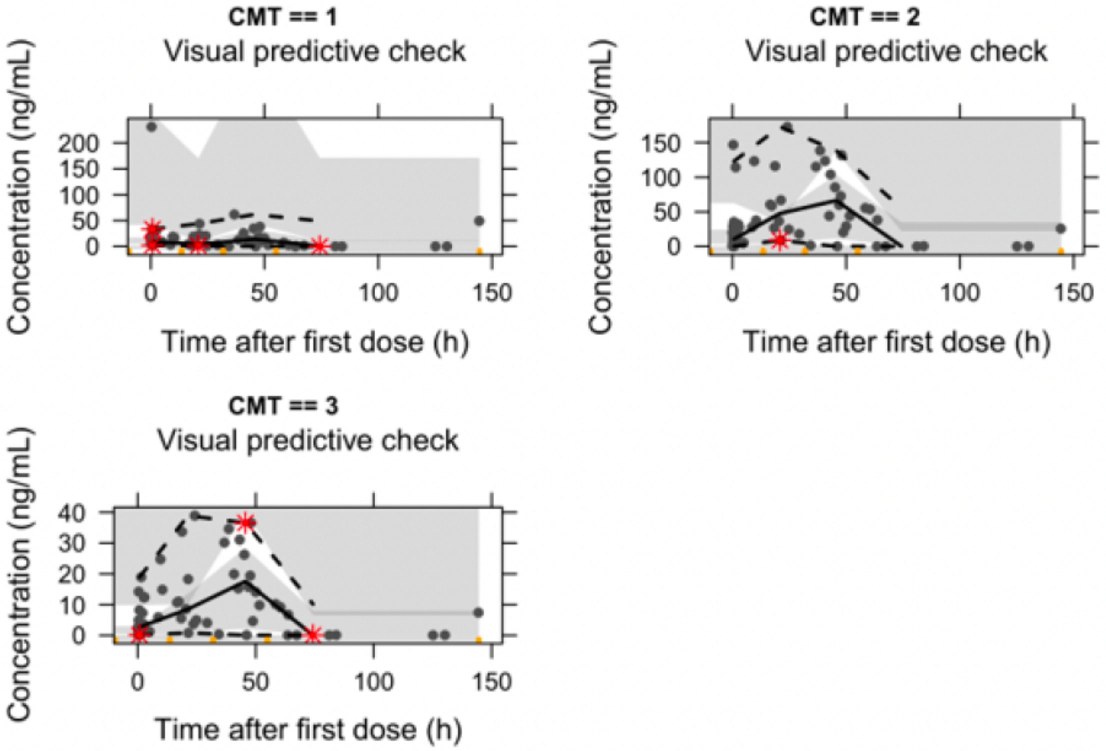

VPC produced using the parameters estimated by the final morphine PK model. The VPC are stratified by compounds: morphine (top left), M3G (top right) and M6G (bottom left). The shaded grey area is the 95 percent prediction interval. The black solid line is the median of the observed data; the black dashed lines are the 5 th and 95 th percentiles of the observed data
