# Supplementary figures and images for: Pharmacokinetic and Pharmacodynamic Modeling of Clonidine and Midazolam for Sedation in Pediatric Intensive Care
Source: Paediatr Anaesth. 2025 Oct 4;35(12):1053–62. doi: 10.1111/pan.70050 (PMC12603884; doi:10.1111/pan.70050)

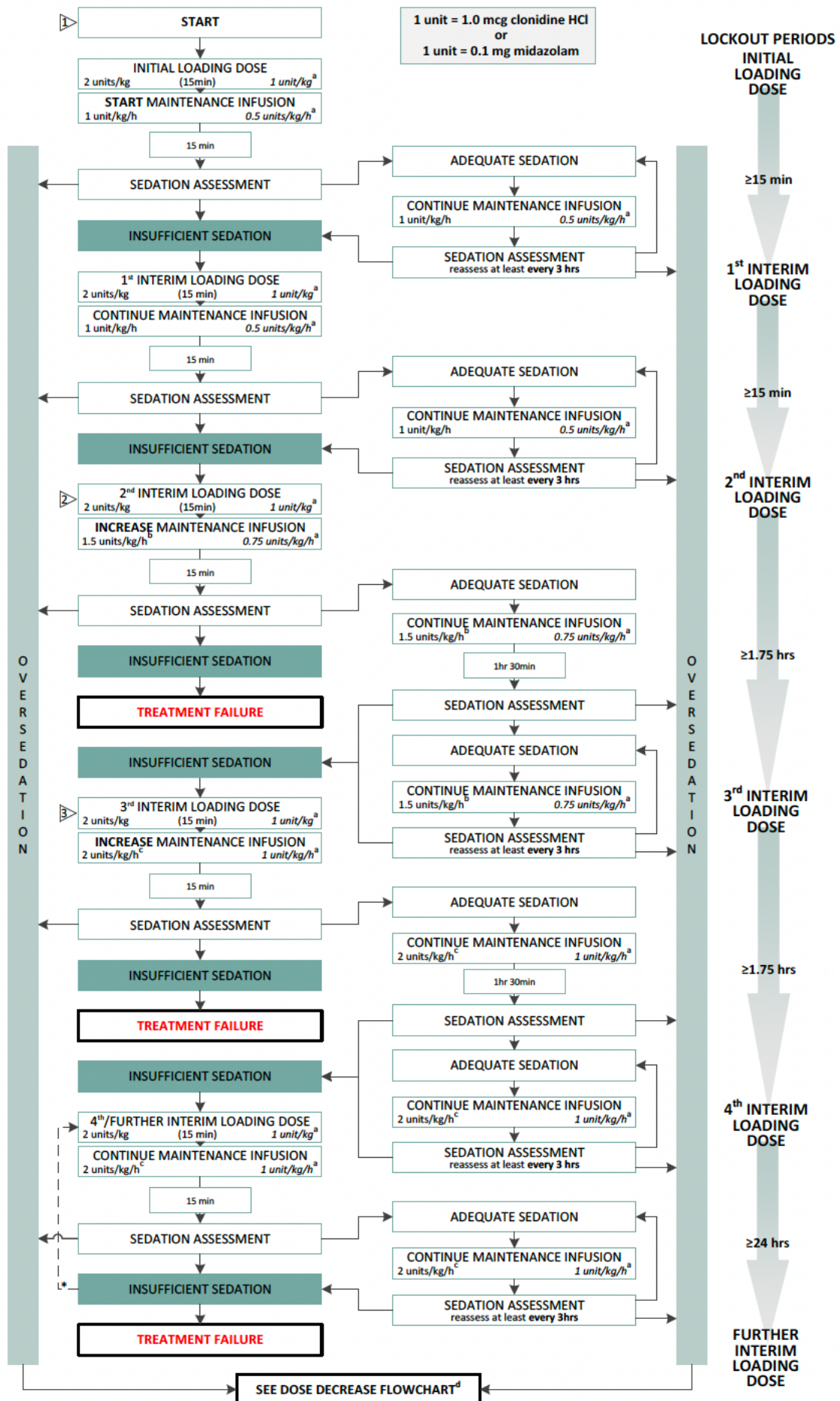

Supplement: Supplementary file 1 — [S1] Primary Endpoint Analysis. [S2] Dosing_Algorithm. [S3] Diagnostic plots clonidine PK model. [S4] Diagnostic plots midazolam PK model. [S5] PKPD observed data. [S6] Parameters estimated using the separate PKPD models. [S7] Nonmem output PKPD model. [S8] Diagnostic plots for final joint PKPD model. [S9] Result PK model morphine. [file PAN-35-1053-s001.zip › Dosing_Algorithm.pdf]

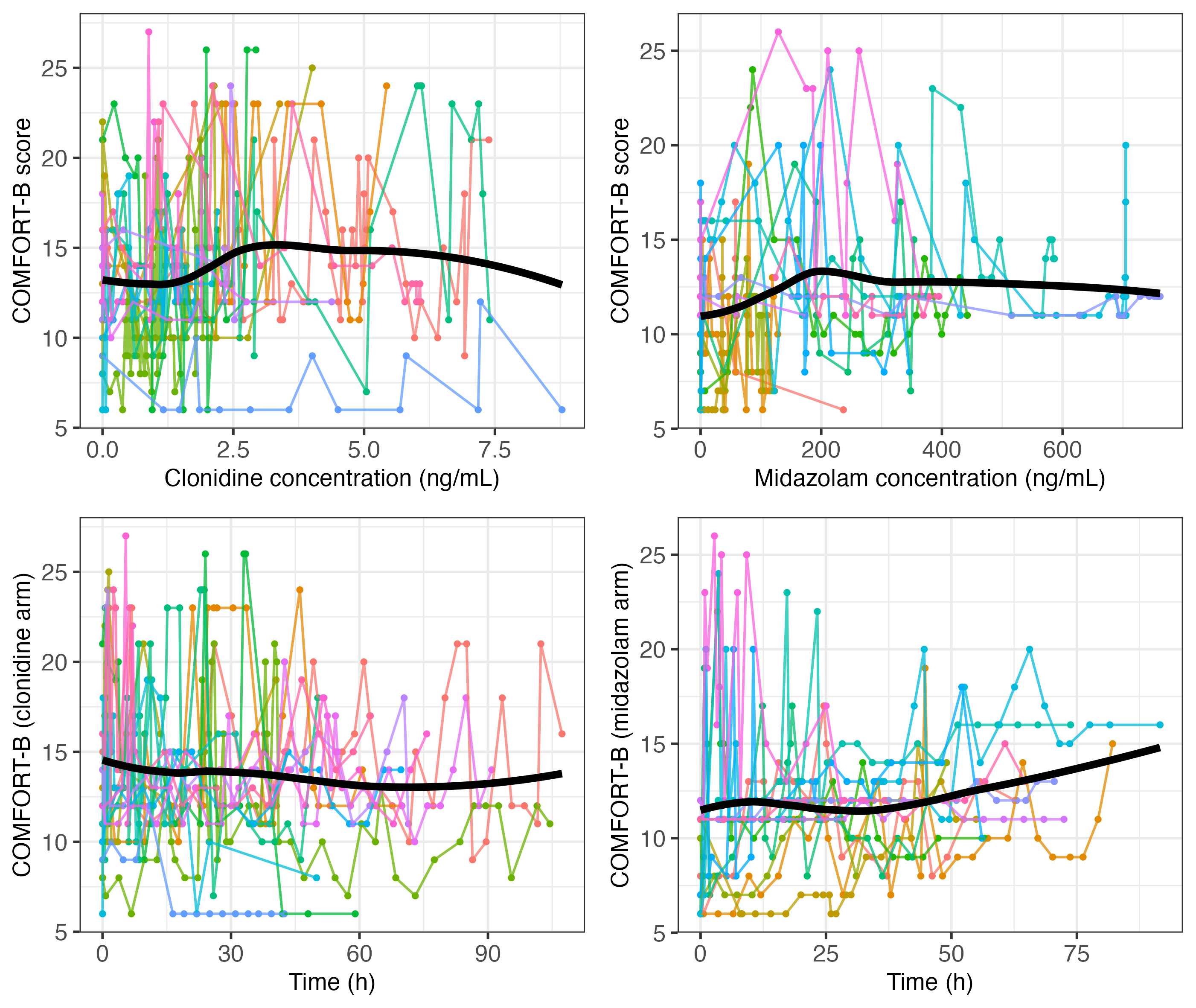

Supplement: Supplementary file 1 — [S1] Primary Endpoint Analysis. [S2] Dosing_Algorithm. [S3] Diagnostic plots clonidine PK model. [S4] Diagnostic plots midazolam PK model. [S5] PKPD observed data. [S6] Parameters estimated using the separate PKPD models. [S7] Nonmem output PKPD model. [S8] Diagnostic plots for final joint PKPD model. [S9] Result PK model morphine. [file PAN-35-1053-s001.zip › PKPD observed data.tiff]
